# Feeding *Brassica* vegetables to rats leads to the formation of characteristic DNA adducts (from 1-methoxy-3-indolylmethyl glucosinolate) in many tissues

**DOI:** 10.1007/s00204-021-03216-8

**Published:** 2022-01-07

**Authors:** Hansruedi Glatt, Wolfram Engst, Simone Florian, Monika Schreiner, Chimgee Baasanjav-Gerber

**Affiliations:** 1 German Institute of Human Nutrition (DIfE), Potsdam-Rehbrücke, 14558 Nuthetal, Germany; 2 Department Food Safety, Federal Institute for Risk Assessment (BfR), Max-Dohrn-Strasse 8-10, 10589 Berlin, Germany; 3 Leibniz Institute of Vegetable and Ornamental Crops (IGZ), 14979 Grossbeeren, Germany

**Keywords:** Broccoli, DNA adducts, Glucosinolates, Neoglucobrassicin

## Abstract

Juices of *Brassica* vegetables are mutagenic and form characteristic DNA adducts in bacteria and mammalian cells. In this study, we examined whether such adducts are also formed in vivo in animal models. Rats fed raw broccoli ad libitum in addition to normal laboratory chow for 5 weeks showed one major adduct spot and sometimes an additional minor adduct spot in liver, kidney, lung, blood and the gastrointestinal tract, as determined by ^32^P-postlabelling/thin-layer chromatography. Adducts with the same chromatographic properties were formed when herring sperm DNA (or dG-3’-phosphate) was incubated with 1-methoxy-3-indolylmethyl glucosinolate (phytochemical present in *Brassica* plants), in the presence of myrosinase (plant enzyme that hydrolyses glucosinolates to bioactive breakdown products). UPLC–MS/MS analysis corroborated this finding: 1-Methoxy-3-indolylmethyl-substituted purine nucleosides were detected in the hepatic DNA of broccoli-fed animals, but not in control animals. Feeding raw cauliflower led to the formation of the same adducts. When steamed rather than raw broccoli was used, the adduct levels were essentially unchanged in liver and jejunum, but elevated in large intestine. Due to inactivation of myrosinase by the steaming, higher levels of the glucosinolates may have reached the large bowl to be activated by glucosidases from intestinal bacteria. In conclusion, the consumption of common *Brassica* vegetables can lead to the formation of substantial levels of DNA adducts in animal models. The adducts can be attributed to a specific phytochemical, neoglucobrassicin (1-methoxy-3-indolylmethyl glucosinolate).

**Supplementary Information:**

The online version contains supplementary material available at 10.1007/s00204-021-03216-8.

## Introduction

Juices of various *Brassica* vegetables have shown mutagenic activity in bacteria and mammalian cells in culture (Kassie et al. 1996; Baasanjav-Gerber et al. 2010; Wiesner et al. 2014). This mutagenicity was associated with the formation of characteristic DNA adducts in the target cells (Baasanjav-Gerber et al. 2010). Likewise, adducts with the endogenous DNA were formed when Brassicaceae vegetables were homogenised and then incubated at 37 °C for 15 min to 16 h (Baasanjav-Gerber et al. 2011a). We demonstrated that these various genotoxic effects are mediated by breakdown products of glucosinolates (GLs) (Baasanjav-Gerber et al. 2011a, b; Wiesner et al. 2014). These findings raised the question whether GLs may also form DNA adducts in vivo after the consumption of brassicaceous crops. To this end, we fed broccoli and cauliflower to rats and then analysed various rat tissues for GL-derived DNA adducts.

This work was conducted some years ago. What is described in the preceding paragraph, reflects the stage of knowledge at the beginning of this study. However, in parallel to this study and afterwards, we performed and published other work on biological activities (genotoxicity, modulation of carcinogenesis, formation of protein adducts) and the bioactivation of GLs, in particular neoglucobrassicin (nGBS, 1-methoxy-3-indolylmethyl glucosinolate) and glucoraphanin (4-methylsulfinylbutyl glucosinolate). This work is outlined at the end of the discussion section under the heading “Epilogue”.

## Materials and methods

### Chemicals and enzymes

Micrococcus nuclease, proteinase K, RNase A and bacterial alkaline phosphatase were purchased from Sigma-Aldrich (Taufkirchen, Germany). Phosphodiesterase II from calf spleen was obtained from Merck Biosciences Ltd. (Darmstadt, Germany), nuclease P1 from MP Biochemicals LLC (Eschwege, Germany), T4 polynucleotide kinase from MBI Fermentas (St. Leon-Rot, Germany), and [γ-^32^P]ATP from Hartmann Analytic (Braunschweig, Germany). GLs and myrosinase (from seeds of *Sinapis alba* L.) were generous gifts of Dr. Renato Iori, Bologna. They were prepared as described elsewhere (Baasanjav-Gerber et al. 2011b). One myrosinase unit was defined as the amount of enzyme able to hydrolyse 1 μmol sinigrin per min at pH 6.5 and 37 °C.

### Plant material

Nine sets of broccoli (*Brassica oleracea* var. *italica*) cv. Marathon and four sets of cauliflower (*Brassica oleracea* var. *botrytis*) cv. Marine were grown at the experimental fields of the Leibniz Institute of Vegetable and Ornamental Crops, Grossbeeren (Germany) in the summer/autumn growing cycle (planting in May till August) and were harvested sequentially from August to November. Fertilisation, irrigation, and plant protection corresponded to the guidelines of the integrated cultivation for broccoli and cauliflower (Winkhoff 1992). After harvest, broccoli and cauliflower were stored at 4 °C and used within 2 weeks in feeding studies. Aliquots of the broccoli and cauliflower used were analysed for their GL contents.

### Animals

All animal experiments were performed with permission of the Landesamt für Verbraucherschutz, Landwirtschaft und Flurneuordnung, Referat 23, of the State of Brandenburg (file number 32-44,456+1). Male Sprague Dawley rats were obtained from the Charles River Laboratory (Sulzfeld, Germany). The animals were housed individually. They were maintained at a day/night cycle of 12 h/12 h at 22 ± 2 °C. We noticed that old animals habituated to standard laboratory chow only consumed modest amounts of broccoli. However, consumption could be strongly enhanced, when weaning animals were accustomed to fresh food. Therefore, 3-week-old rats received green salad, cucumber and apple in addition to standard rat diet (V1534-000, ssniff, Soest, Germany) ad libitum for a few weeks before the start of the actual experiment.

### Feeding regimen

*Experiment I: feeding of raw broccoli:* When the rats were 6 weeks old and weighed about 200 g, the feeding of broccoli was started. Five rats were now fed raw broccoli and standard rat diet, both ad libitum, for 5 weeks. Broccoli was bought from local stores for the initial three weeks. In the last 2 weeks, we used broccoli grown as described in the section headed “Plant material”. One further rat (“control”) only received standard diet. The body weight of the rats (Table S1, with S referring to Supporting Information) and the amount of the standard rat chow consumed (Table S2) were measured weekly, whereas the amount of broccoli consumed was determined daily (Table S3). In the last 24 h before killing, the rats were only fed broccoli. They were killed by decapitation. Mucosa from small and large intestinal tissues was scraped off with a spatula and then frozen in liquid nitrogen. Other tissues were washed in isotonic saline and then frozen in liquid nitrogen immediately. Whole blood (5–8 ml per animal) was collected in 9-ml S-Monovette (Sarstedt, Nümbrecht, Germany) tubes containing EDTA (final concentration 1–2 mg/ml). Then, buffy coats were obtained by centrifugation at 1800 g and 4 °C for 10 min. The buffy coat and the tissues were stored at − 80 °C until DNA isolation.

*Experiment II: feeding of three different* Brassica *diets:* When the rats were about 8 weeks old, they were divided into four groups (6 animals per group) receiving different diets for the next 5 weeks: the control group (group 1, rats 1–6) only received standard laboratory chow. The other animals received raw broccoli (group 2, rats 7–12), steamed broccoli (group 3, rats 13–18) or raw cauliflower (group 4, rats 19–24) ad libitum in addition to the standard laboratory chow*.* To prevent the leakage of GLs into the water, we steamed the broccoli (15 min, 100 °C) rather than cooking it in water. The body weight of the rats (Table S6) and the amount of standard diet consumed (Table S7) were measured weekly, whereas the amount of *Brassica* vegetable consumed was determined daily (Table S8). In the last 24 h before being killed, the rats were only fed *Brassica* vegetable in the corresponding groups. Animals were killed and tissues were collected as described for experiment I.

### Analysis of GLs

Fully developed broccoli or cauliflower heads were used in the feeding study. A mixed sample of florets (inflorescences with a diameter of 5 ± 1 cm) from five broccoli or cauliflower heads was taken at each harvest date for GL analysis. For this analysis, the material (200 g fresh matter) was immediately deep-frozen (− 28 °C), then freeze-dried and finely ground. For the further sample preparation and the high-performance liquid chromatography (HPLC) analysis, we followed the protocol published by Krumbein et al. (2005). Duplicates of freeze-dried sample material (0.5 g) were heated to and incubated at 75 °C for 1 min, extracted with 4 ml of a methanol/water mixture (v/v = 7:3, *t* = 70 °C), and then, after adding barium acetate solution (1 ml, 0.4 M), centrifuged at 1800 g for 10 min. For an internal standard, 200 µl of a 5-mM stock solution of 2-propenylglucosinolate (sinigrin) in methanol was added to one of the duplicates just before the first extraction. The residue was extracted twice more with 3 ml of the methanol/water mixture (v/v = 7:3, *t* = 70 °C). The supernatants were pooled and made up to 10 ml with the methanol/water mixture (v/v = 7:3, t = 70 °C). A 5-ml aliquot of this extract was applied to a 250-µl DEA-Sephadex A-25 ion-exchanger (acetic acid-activated, Sigma-Aldrich) and rinsed with 10 ml bi-distilled water. Next, 250 µl of a purified solution of aryl sulphatase (at least 0.5 U/ml) (Boehringer-Mannheim GmbH, Mannheim, Germany) was applied and left for 12 h, before the desulphated compounds were flushed with 5 ml bi-distilled water. The analysis of the desulphated GLs was conducted by HPLC (Merck Hitachi, Darmstadt, Germany) using a Spherisorb ODS2 column (5 µm, 250 × 4 mm, Waters, Milford, USA). A gradient of 0–20% acetonitrile in water was selected from 2 to 34 min, followed by 20% acetonitrile in water until 40 min, and then 100% acetonitrile for 10 min until 50 min. The flow rate was 1.3 ml/min. Levels of desulphated GLs were determined at a wavelength of 229 nm using a diode-array detector. Their structure was identified by HPLC-APCI-MS/MS using Agilent 1100 series (Agilent Technologies, Walbronn, Germany) in the positive ionisation mode (Zimmermann et al. 2007). GL concentrations were calculated using 2-propenylglucosinolate as internal and external standard and using the response factor of each compound relative to the standard (EEC Regulation 1864/90,1990). Determination of GLs was performed in duplicate. In general, the variation was less than 5% of the mean.

### DNA extraction from tissues and blood

DNA was isolated from tissues using a procedure of Gupta (1984) with some modification. One gram of frozen tissue was thawed in 3 ml of 10 mM EDTA (pH 8) and homogenised with a Polytron homogenizer. The homogenate was incubated with 30 µl of proteinase K (10 mg/ml) and 500 μl of sodium dodecyl sulphate (10% in water) at 37 °C for 1 h. After the addition of 0.5 ml of 1 M Tris–HCl (pH 7.4) and 10 μl RNAse A (10 μg/μl), the homogenate was further incubated at 37 °C for 30 min. Thereafter, the DNA was extracted in three steps. In each step, one volume (5.04 ml) of a different solvent was used—phenol, phenol:chloroform:isopropanol (25:24:1, v/v/v) and chloroform:isopropanol (24:1, v/v). The extractions were performed in 15-ml Falcon tubes. The phases were separated by centrifugation at 11,000 *g* and 4 °C for 10 min. After the last extraction, the DNA was precipitated by the addition of the 0.1 volumes of 3 M sodium acetate and 1.5 volumes of ice-cold ethoxyethanol. After keeping the samples at room temperature for 30 min, DNA pellets were obtained by centrifugation at 9500 *g* and 4 °C for 10 min. The DNA was dissolved in citrate solution (0.15 mM sodium citrate, 1.5 mM NaCl, pH 7.5).

The buffy coat was thawed in 10 ml of red blood cell lysis buffer (10 mM Tris–HCl pH 7.6, 5 mM MgCl_2_, 10 mM NaCl) and then centrifuged at 900 *g* and 4 °C for 10 min. The resulting supernatant was discarded and the pellet was resuspended in 3 ml of white cell lysis buffer (10 mM Tris–HCl pH 7.6, 10 mM EDTA pH 8.0) with addition of 50 μl of 10% sodium dodecyl sulphate and 50 μl of proteinase K (10 mg/ml). The mixture was gently shaken horizontally with 50 rpm at 42–50 °C overnight. On the next day, the DNA was extracted using the same protocol as for DNA from solid tissues.

### ^32^P-postlabelling analysis of DNA adducts

The ^32^P-postlabelling method was conducted using a nuclease-P1-enrichment step as described elsewhere in detail (Baasanjav-Gerber et al. 2011a). For preparing reference adducts of nGBS, 1 μM nGBS (generously provided by R. Iori, Bologna) was incubated with herring sperm DNA (1 mg/ml) in the presence of the myrosinase (6 mU) in a total volume of 100 µl sodium phosphate buffer (10 mM, pH 7.4) at 37 °C for 2 h. The DNA was precipitated by adding 10 µl of NaCl (5 M) and 60 µl of isopropanol. This reference DNA was digested and labelled using the same protocol as for tissue DNA (Baasanjav-Gerber et al. 2011a). Adduct levels were determined from the radioactivity located in the adduct spots, the specific radioactivity of [γ-^32^P]ATP used and the labelling efficiency of the batch of T4 polynucleotide kinase (using 3’-phosphoadenosine as a reference substrate) (Baasanjav-Gerber et al. 2011a). In co-chromatography experiments, the labelled digests of reference DNA and tissue DNA were applied in a 1:10 ratio onto the same thin-layer chromatography plate. The ^32^P-postlabelling method involves various enzymatic steps, whose efficiency may vary for different adducts. Therefore, results are expressed as “relative adduct levels” (RAL) (Phillips and Arlt 2007). In general, the true adduct level is higher due to incomplete recovery.

### UPLC–MS/MS analysis of DNA adducts

**Scheme 1.**
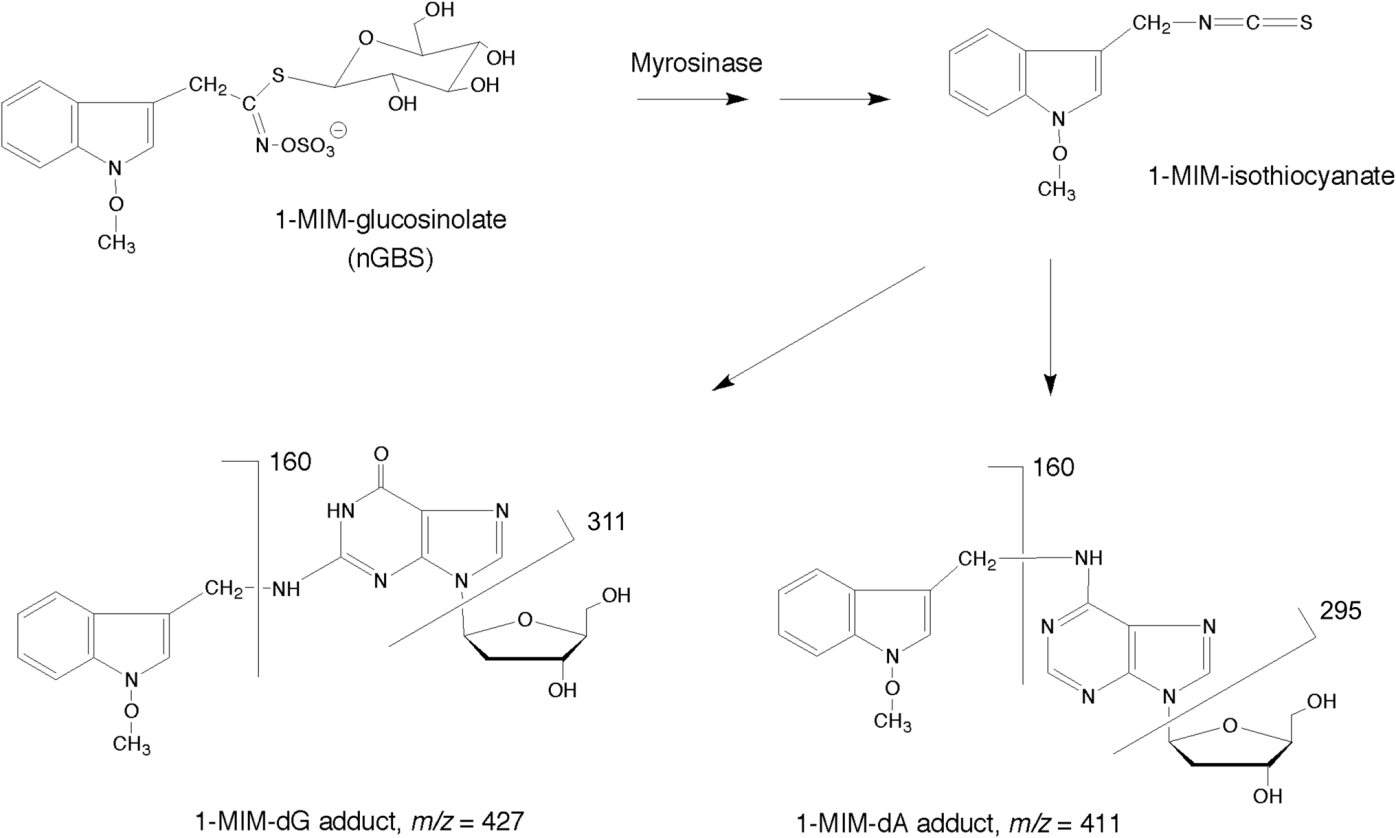
Putative activation pathway of nGBS (1-MIM-GL) and structures of adducts formed in tissues of rats fed *Brassica* vegetable. 1-MIM isothiocyanate is a short-lived intermediate formed from nGBS in the presence of myrosinase (Hanley et al. 1990). Although it is not available for direct investigations, it is a primary candidate for the DNA-forming product, as other breakdown products—1-MIM-alcohol and 1-MIM-nitrile—were much less genotoxic than nGBS activated by myrosinase (Glatt et al. 2011). The numbers given in the panels of the nucleoside adducts indicate *m/z* of the protonated parent molecules and the cationic daughter ions observed. The site of the nucleobases binding to the 1-MIM residue was later verified via synthesis of the standards and NMR analysis (Schumacher et al. 2012)

Rat liver DNA (250 µg) was hydrolysed by incubation with micrococcal nuclease (120 mU) and spleen phosphodiesterase II (2 mU) in succinate buffer (16.7 mM sodium succinate, 8.3 mM CaCl_2_, pH 6.0, total volume 180 µl) at 37 °C for 4 h. Then, 96 µl of 0.5 M Tris buffer (pH 10.5) and 50 µl of bacterial alkaline phosphatase (5 U) were added. After overnight incubation at 37 °C, the sample was diluted by adding 674 µl of water, yielding a total volume of 1.0 ml. It was loaded on a solid-phase extraction column (Bakerbond Octadecyl 7020–03, J. T. Baker, Deventer, the Netherlands) that had been conditioned with methanol and water. After loading, the column was washed with water (twice 500 µl) and eluted with 1.5 ml methanol. The eluate was concentrated in a Speed Vac. The residues of eight equivalent incubations were pooled, dissolved in 50 µl methanol and then analysed by ultra-performance liquid chromatography (UPLC) coupled with tandem mass spectrometry (MS/MS) in the multiple reaction monitoring (MRM) mode. The analyses were carried out on a tandem-quadrupole mass spectrometer (Quattro Premier XE) interfaced with an Acquity UPLC System (both from Waters). The separation was conducted on an Acquity BEH Phenyl column (1.7 µm; 2.1 × 100 mm, Waters), which was kept at 25 °C. The samples were cooled to 5 °C, and aliquots of 4 µl were given onto the column. Elution was performed using gradients of A (0.25% formic acid/0.25% acetic acid/99.5% water) and B (0.25% formic acid/0.25% acetic acid/99.5% methanol). The flow rate was 0.3 ml/min. The gradient was used as follows: 2 min isocratic 96% A, linear gradient to 10% A within 2 min, followed by reconditioning to 96% A. The MS/MS analyses of the 1-MIM-2’-deoxynucleoside adducts were performed in the positive electrospray mode. The collision-induced dissociation involved argon as the collision gas at 3.5 × 10^–3^ mbar. Other parameters were: capillary voltage 1.5 kV, cone voltage 17 V, source temperature 110 °C, desolvation temperature 450 °C, and desolvation gas (N_2_) 950 l/h. The collision energy was attuned to 15 eV. MRM analyses of two transitions of each adduct were recorded (MIM-dA: 411.2 → 295.2 and 411.2 → 160.2; MIM-dG: 427.2 → 311.2 and 427.2 → 160.2; MIM-dC: 387.2 → 271.2 and 387.2 → 160.2). These transitions correspond to the loss of deoxyribose and the entire nucleoside without the adducted moiety, as illustrated in Scheme 1.

## Results

### Consumption of broccoli or cauliflower, intake of GLs and body weight development

The rats consumed 36 g broccoli per day and animal in the first experiment on average (details in Table S3). In the second experiment, the average daily *Brassica* consumption amounted to 48 g for raw broccoli, 47 g for steamed broccoli and 39 g for cauliflower (Table S8). GL levels in broccoli and cauliflower were regularly monitored (Tables S4 and S9), allowing calculation of their total intake in the experiments (Table 1; details in Tables S5, S10 and S11). The body weight development was similar in *Brassica*-fed and control rats (Tables S1 and S6).

**Table 1:** Intake of total GLs and of nGBS in the different experimental groups receiving *Brassica*-containing diets

Experimental group	Mean intake per day, mg per animal, mean (lowest–highest) of n rats	
	Total GLs	nGBS
Experiment I, raw broccoli (*n* = 5)	13.7 (8.5–25.8)^a^	2.4 (1.4–4.4)^a^
Experiment II, raw broccoli (*n* = 6)	12.8 (7.8–15.5)	2.1 (1.3–2.6)
Experiment II, steamed broccoli (*n* = 6)	13.1 (8.8–16.9)	2.0 (1.4–2.7)
Experiment II, raw cauliflower (*n* = 6)	4.0 (2.1–7.2)	0.42 (0.22–0.79)

For each animal, the mean daily intake (details in Tables S5, S10 and S11) was calculated from the amount of vegetable consumed (Tables S3 and S8) and the GL levels in these materials (Tables S4 and S9)

^a^GL levels were only determined in the broccoli fed during the last two weeks. For the calculation of the total GL intake, it was assumed that the levels were similar in the broccoli used in the initial 3 weeks

### DNA adduct formation in tissues of rats fed broccoli or cauliflower

**Fig. 1.**
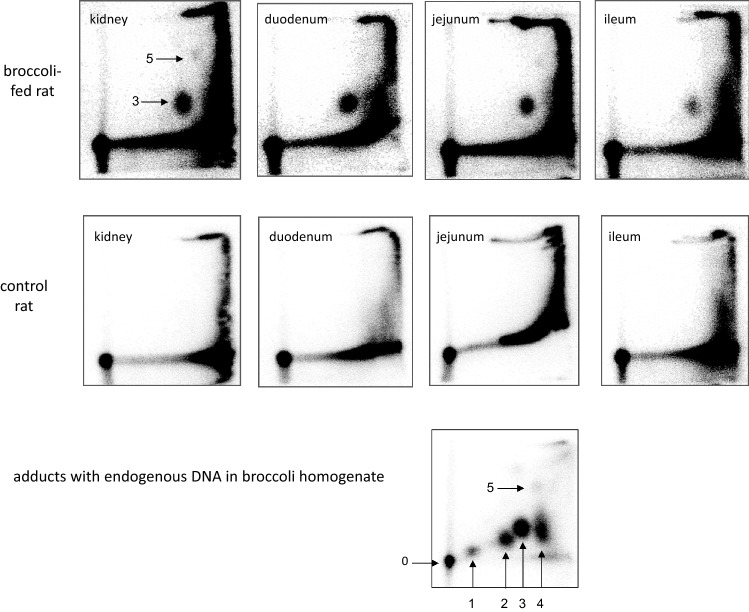
^32^P-Postlabelling/thin-layer chromatographic analysis of DNA from tissues of rats fed raw broccoli (upper row) and control animals (middle row), and of endogenous DNA from broccoli homogenate incubated at 37 °C for 2 h (lowest panel). Adducts in the broccoli homogenate were studied as described in detail elsewhere (Baasanjav-Gerber et al. 2011a). The spots were arbitrarily numbered (0, site of sample application; 1–5, adduct spots). The data presented for rat tissues are from experiment I. However, the autoradiograms are representative for all animals either receiving control or *Brassica*-supplemented diets in both experiments conducted. Adduct levels, calculated from the radioactivity in the marked spots, are presented in Tables 2 and 3. The signals at the edges of the chromatograms represent reagents and site products of the labelling reaction

^32^P-postlabelling analyses demonstrated the presence of one or two DNA adduct spots in 11 out of 13 tissues isolated from rats fed raw broccoli in experiment I (upper row of Fig. 1, Table 2). These adducts were absent in control animals (middle row of Fig. 1). Their chromatographic properties were similar to adduct spots 3 and 5 detected in the endogenous DNA of broccoli homogenate (lower row of Fig. 1).

**Table 2:** DNA adduct levels in different tissues of rats fed raw broccoli and standard chow, both ad libitum, for 5 weeks—results from experiment I

Tissue	DNA adducts/10^8^ nucleotides (RAL)
Liver	39 ± 20
Kidney	37 ± 16
Lung	20 ± 7
Glandular stomach	21 ± 16
Duodenum, mucosa	28 ± 10
Jejunum, mucosa	31 ± 16
Ileum, mucosa	12 ± 3
Caecum, mucosa	11 ± 1
Proximal colon, mucosa	8 ± 3
Distal colon, mucosa	3 ± 2
White blood cells	13 ± 8
Thymus	< LOD ^a^
Prostate	< LOD ^a^

Adducts were detected by the ^32^P-postlabelling/thin-layer chromatography method. Representative chromatograms are shown in Fig. 1. Adduct levels were calculated from the radioactivity contained in spots 3 and 5. They are means ± SD of 5 rats. The control rat, receiving only standard chow, showed no adduct spots

^a^The limit of detection was 2 adducts/10^8^ nucleotides

In the repeat experiment, two additional feed groups were used, one received steamed broccoli, the other received raw cauliflower. In this experiment, only four tissues were analysed for the presence of adducts, as the ^32^P-postlabelling analysis is very labour-intensive. All these tissues showed adduct spot 3 and sometimes also adduct spot 5 in each individual animal fed *Brassica* crops (Table 3); again, the adducts were absent in control rats. The adduct levels obtained in the raw-broccoli group in the gut were similar in both experiments. The hepatic adduct levels obtained in the raw-broccoli group in experiment II were somewhat higher than in experiment I (*cf.* Tables 2 and 3), although the total intake of GLs was similar (Table 1). However, profiles and levels of GLs differently varied over the feeding periods (Tables S4 and S9). Moreover, somewhat older animals were used in experiment II than in experiment I.

**Table 3:** DNA adduct levels in tissues of rats fed *Brassica* vegetable and standard chow, both ad libitum, for 5 weeks—results from experiment II

Tissue	DNA adducts/10^8^ nucleotides (RAL)		
	Group 2 (raw broccoli)	Group 3 (steamed broccoli)	Group 4 (raw cauliflower)
Liver	52 ± 13	56 ± 19	27 ± 19
Jejunum, mucosa	28 ± 12	32 ± 15	17 ± 6
Caecum, mucosa	9 ± 3	27 ± 21	6 ± 5
Colon, mucosa	7 ± 5	22 ± 16	5 ± 2

Adducts were detected by the ^32^P-postlabelling/thin-layer chromatography method. Adduct levels were calculated from the radioactivity contained in spots 3 and 5. They are means and SD of six rats. The control rats (group 1), only receiving standard chow, showed no adduct spots (with a limit of detection of 2 adducts per 10^8^ nucleotides)

Feeding of raw cauliflower led to the formation of the same adduct spots as feeding of raw broccoli, but adduct levels were somewhat lower—by a factor of 1.4 to 1.9 depending on the tissue (Table 3). This compares to a 3.2-fold lower intake of total GLs in the raw cauliflower compared to the raw-broccoli group (Table 1). However, profiles of GLs were somewhat different in these two varieties of *Brassica oleracea* (Table S9). When steamed rather than raw broccoli was used (Table 3), adduct levels were unchanged in the jejunum and the liver, but were enhanced nearly threefold in large bowl (Wilcoxon rank test *p* = 0.02 and 0.05 for caecum and colon mucosa, respectively). From previous work, we know that the steaming procedure used strongly reduces myrosinase activity in broccoli, as indicated by a decrease in the adduct formation in the homogenate by 90% (Baasanjav-Gerber et al. 2011a). Thus, it is likely that higher levels of GLs reached the large intestine when steamed rather than raw broccoli was used. There they were either activated by the residual myrosinase activity from broccoli (taking advantage from the long transit period in this part of the gut) or by glucosidases from intestinal bacteria.

### Characterisation of the adducts in rats fed broccoli

Previously, we demonstrated that adduct spot 3 in homogenates of brassicaceous plant material co-migrates with adducts formed in herring sperm DNA by a specific GL, nGBS (structure presented in Scheme 1) (Baasanjav-Gerber et al. 2011a, b). Using a total of eleven other GLs, no adduct was detected with comparable chromatographic properties (Baasanjav-Gerber et al. 2011b). In contrast, several different GLs, including nGBS, formed adducts with the chromatographic properties of spot 5. In subsequent experiments, nGBS and myrosinase were incubated with individual dN-3’-phosphates rather than DNA. The major adducts formed with dG- and dA-3’-phosphate migrated as the adduct spots 3 and 5, respectively (data not shown).

**Fig. 2:**
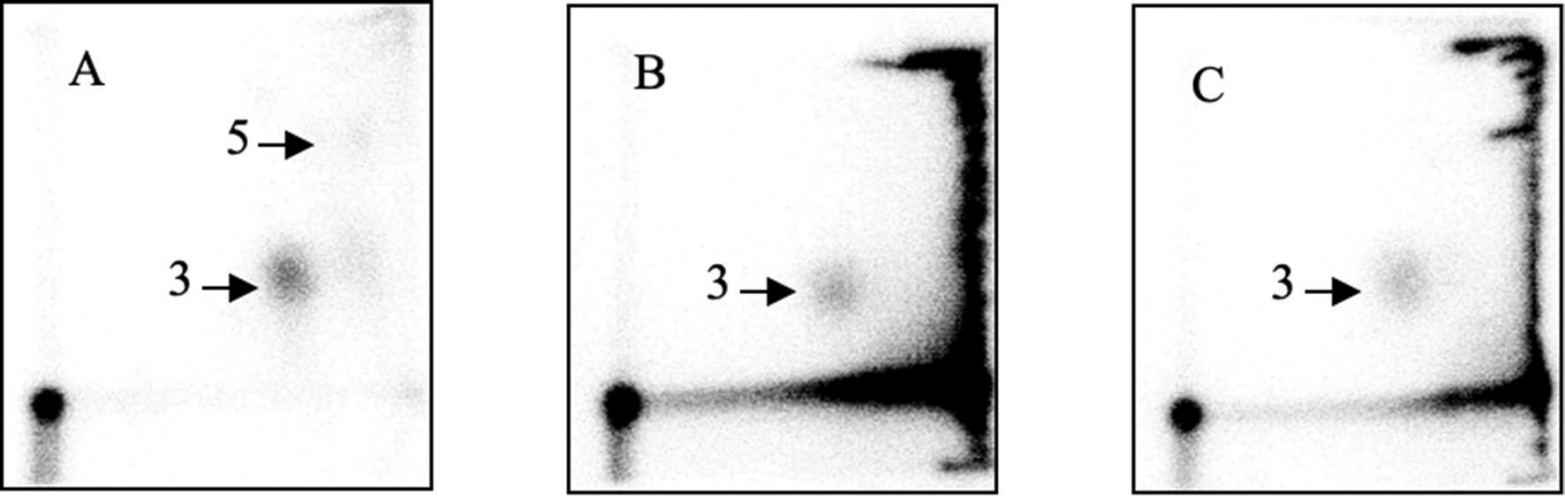
Co-chromatography of adducts detected in a rat fed broccoli and of adducts formed by nGBS in vitro. **A** Herring sperm DNA treated with nGBS in the presence of myrosinase (adduct level in spot 3: 96 ± 7 per 10^8^ nucleotides, mean and range of two analyses); **B** liver DNA from a broccoli-fed rat (adduct level in spot 3: 13 ± 2 per 10^8^ nucleotides); **C** 1:9 mixture of nGBS-treated herring sperm DNA and liver DNA from the broccoli-fed rat. This ratio was chosen to obtain similar contributions from both DNA samples to spot 3. Therefore, the expected adduct level (per 10^8^ nucleotides) in this sample was 21.3 (9.6 from the reference material and 11.7 from the hepatic DNA), which nearly matched the actual observed adduct level (28 ± 5 per 10^8^ nucleotides)

Using co-chromatography techniques, we verified that the major adducts found in liver of a rat fed raw broccoli exactly chromatographs as the 1-MIM-dG adduct (Fig. 2). The signal of the second adduct was too faint for co-chromatography experiments.

**Fig. 3:**
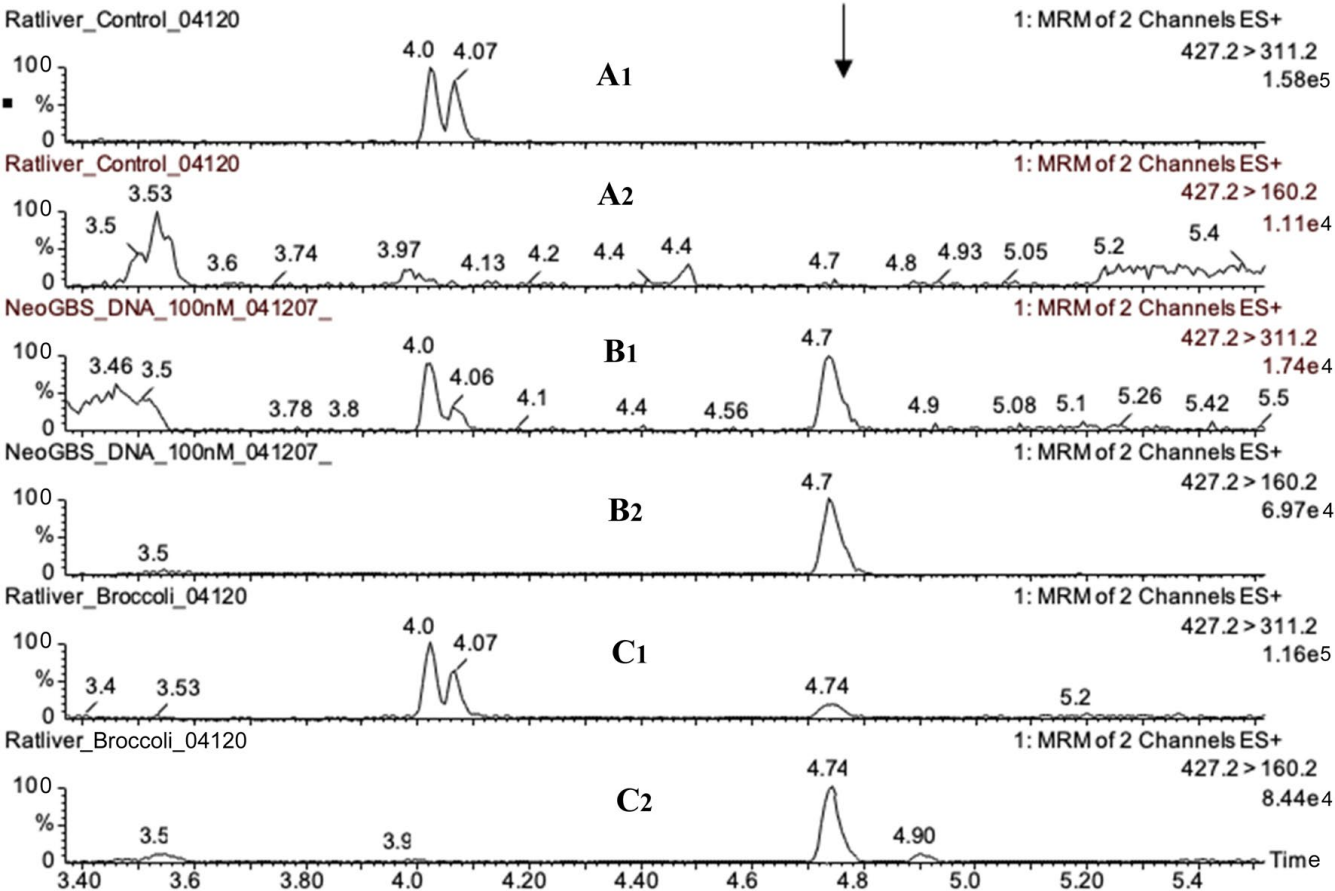
Presence of the 1-MIM-dG adduct in a rat fed broccoli, as demonstrated by UPLC–MS/MS analysis. Hepatic DNA was isolated from a control rat only receiving standard chow (**A**) and an animal additionally receiving raw broccoli ad libitum (**C**). Both animals were from experiment 1. Moreover, herring sperm DNA was incubated with nGBS in the presence of the myrosinase (**B**). The DNA samples were digested into nucleosides, and then analysed by UPLC–MS/MS with positive electrospray ionisation and recording of the 427.2 → 311.2 and 427.2 → 160.2 transitions. Protonated MIM-dG has an *m/z* ratio of 427, as illustrated in Scheme 1. The daughter ions with *m/z* 311 (recorded in traces in A1, B1, C1) and 160 (recorded in traces A2, B2, C2) result from loss of dG and 2’-deoxyribose, respectively (Scheme 1). Since the strongest signal in each trace was set as 100%, the ordinate scales vary. The critical signal (retention time of 4.7 min) is marked by an arrow. This signal was absent in the control rat

**Fig. 4:**
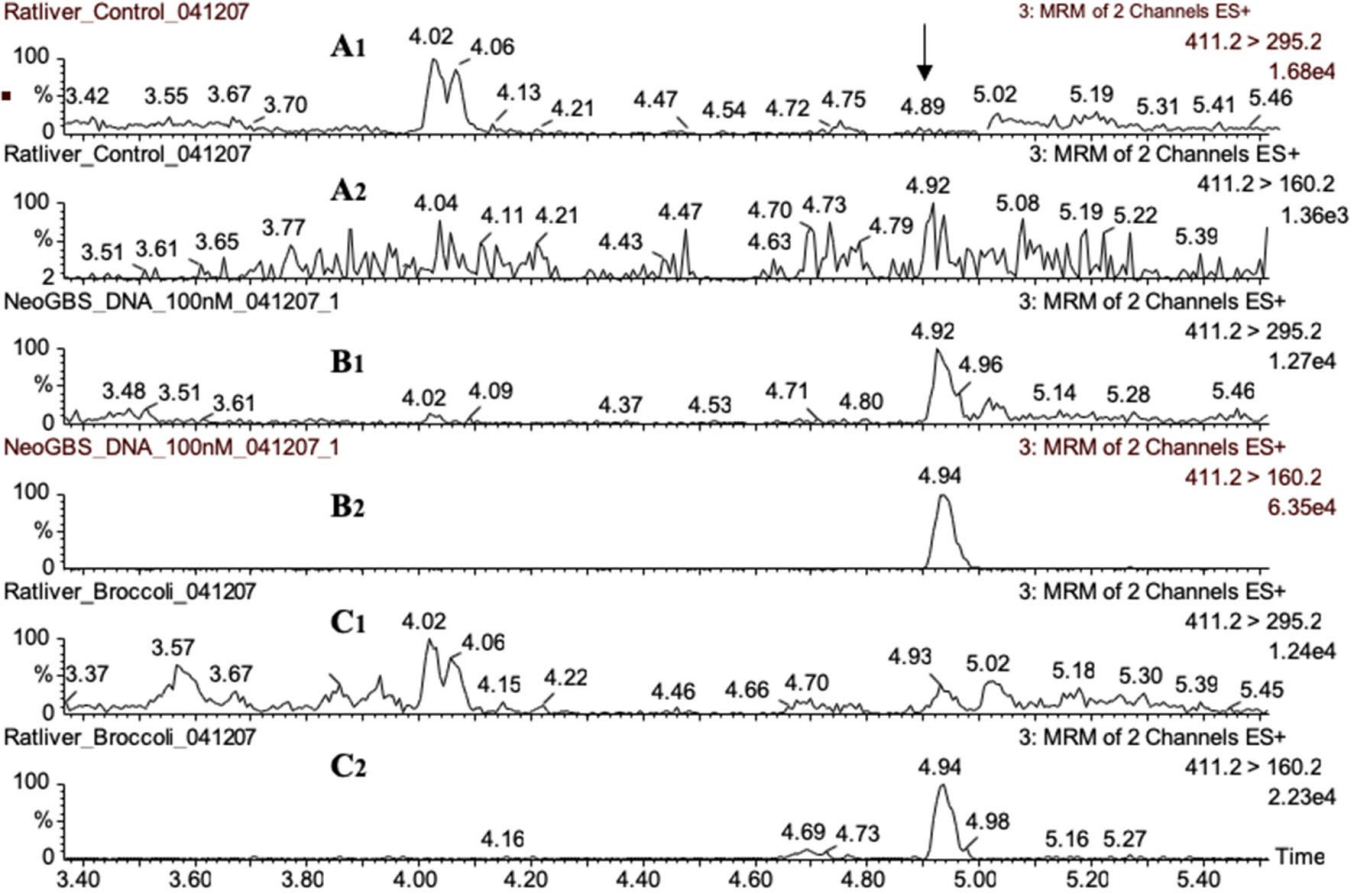
Presence of the MIM-dA adduct in a rat fed with broccoli, as demonstrated by UPLC–MS/MS analysis. For experimental details, see legend to Fig. 3. However, other transitions were recorded: 411.2 → 295.2 and 411.2 → 160.2. Protonated 1-MIM-dA has an *m/z* of 411, as illustrated in Scheme 1. The daughter ions with *m/z* 295 (recorded in traces in A1, B1, C1) and 160 (recorded in traces A2, B2, C2) result from loss of 2’-deoxyribose and dA, respectively (Scheme 1). The critical signal (retention time of 4.92–4.94 min) is marked by an arrow. In the control rat, the 411.2 → 295.2 and 411.2 → 160.2 signals were absent and ten times lower than in the broccoli-fed rat, respectively

Using mass-spectrometric method, we demonstrated that 1-MIM-dG, 1-MIM-dA and 1-MIM-dC adducts were formed, when nGBS was incubated with herring sperm DNA in the presence of myrosinase (Schumacher et al. 2012). The *m/z* ratios of the protonated dG and dA adducts as well as the major daughter ions are shown in Scheme 1. Two transitions were used for the UPLC–MS/MS analyses of each adduct. These analyses clearly demonstrated the presence of MIM-dG (Fig. 3) and MIM-dA (Fig. 4) adducts in hepatic DNA of broccoli-fed rats, but not in DNA of control rats. The MIM-dC adduct was not detected (data not shown) in vivo, which was not surprising in as much as the level of this adduct was very low in reference DNA reacted with activated nGBS (Schumacher et al. 2012).

### Intake of nGBS

After demonstrating that nGBS is involved in the formation of DNA adducts that were observed in rats fed *Brassica* vegetable, we calculated the intake of this GL in the different experimental groups, taking into account the consumption levels and the nGBS content in the different materials (Table 1, details in the Supporting Information). The mean daily nGBS intake ranged from 1.3 to 4.4 mg in the 17 rats receiving broccoli-containing diets and from 0.22 to 0.79 mg in the six rats receiving the cauliflower-containing diet (Table 1). The lower intake from cauliflower was reflected in lower DNA adduct levels (Table 3).

## Discussion

In this study, we demonstrated that feeding raw and steamed *Brassica* vegetable to rats leads to the formation of characteristic DNA adducts that are absent in control rats. The adducts were detected by ^32^P-postlabelling/thin-layer chromatography (Phillips and Arlt 2007) as well as UPLC–MS/MS (Tretyakova et al. 2012).

The ^32^P-postlabelling method detects a wide range of different adducts, as long as their nucleoside 3’-phosphates are substrates for the T4 polynucleotide kinase and the resulting nucleoside 3’,5’-biphosphates can be separated chromatographically from unmodified nucleoside 3’,5’-biphosphates (Phillips and Arlt 2007). In particular, knowledge of the structure of an adduct is not required for its detection by ^32^P-postlabelling. Using this method, various adduct spots have been detected in tissues of animals not deliberately exposed to mutagens (Randerath et al. 1995, 1996). These adduct spots, also termed I-compounds, primarily occur in old animals. It is suspected, and has been confirmed in some cases, that they are produced by reactive intermediates from the endogenous metabolism and food-borne genotoxicants. However, we did not find any adduct spots in tissues of control animals. This negative finding may be due to the fact that we used young animals. It implies that the level of adducts induced by the *Brassica* diets strongly exceeded the normal I-compound level of animals of this age.

The ^32^P-postlabelling method as such does not provide any appreciable information on the structure of an adduct detected. However, chromatographic comparison with adduct standards can support or reject hypotheses on the structure. Thus, the major adduct formed in animals fed *Brassica* vegetable co-chromatographed with a dG adduct generated by a breakdown product of nGBS, but not with any other GL-derived adduct known. Likewise, the chromatographic properties of the minor adduct in these animals were similar to those of a dA adduct formed by a breakdown product of the same GL, nGBS.

Theoretically, UPLC–MS methods can be used to detect adducts of unknown structure in DNA digested to nucleosides (Guo et al. 2017; Tretyakova et al. 2012). Although untargeted adduct searches by UPLC–MS is continuously improving, their sensitivity is still below that of the ^32^P-postlabelling technique. However, the sensitivity of UPLC–MS methods can be strongly enhanced when hypotheses on the masses of the adduct and daughter ions can be proposed or when the mass spectrometer can be tuned with reference materials. In the present study, we optimised the analytical conditions for reference adducts formed by nGBS with dG, dA and dC in the presence of myrosinase. Using the standards, we clearly could demonstrate the presence of these dG and dA adducts in the hepatic DNA of rats fed broccoli, based on identical retention time, molecular mass of the protonated adduct, and characteristic fragmentations occurring in a similar ratio for the sample and the standard adduct. The dC adduct, being formed only at a very low level in vitro (Schumacher et al. 2012), was not detected in vivo*.*

Only two adduct spots were observed in animal tissues, whereas five spots occurred in homogenates of broccoli (Fig. 1) and cauliflower (Baasanjav-Gerber et al. 2011a). The adduct spots found in vivo correspond to adducts formed by nGBS. The remaining three adduct spots in the homogenate appear to be formed by other indole GLs, primarily by glucobrassicin (GBS, 3-indolylmethyl glucosinolate) (Baasanjav-Gerber et al. 2011a, b). Although GBS and nGBS form similar levels of DNA adducts in cell-free systems, GBS is much less mutagenic and forms much lower adduct levels in cellular systems than nGBS (Baasanjav-Gerber et al. 2011b). Hanley et al. (1990) detected a short-lived isothiocyanate intermediate in the myrosinase-mediated hydrolysis of nGBS to its carbinol. By contrast, the congeneric isothiocyanate was not found, when GBS was used as the substrate, which was attributed to even higher chemical reactivity. The difference in reactivity was explained by the electron-donating character of the methoxy group (present in nGBS and its decomposition products), deactivating the benzylic carbon atom. An adequately long half-life time is important for penetrating cells and it may be even more important in animal models, where transfer over longer distances is required, than in bacterial and cell culture models.

The detection of MIM DNA adducts in animal models raises the question whether these adducts can induce mutations and cancer in vivo. nGBS activated by myrosinase was strongly mutagenic in bacteria and mammalian cells in culture, where it formed the same DNA adducts (Baasanjav-Gerber et al. 2011b; Glatt et al. 2011) as detected in the present study in rats consuming *Brassica* vegetable. Therefore, we expect that mutations will also be induced in vivo, except in tissues where adducts are exclusively formed in terminally differentiated cells. However, the fact that adducts were detected in 11 out of 13 tissues investigated suggests that the adducts occur in many different cell types. It is unlikely that all of them are unable to fix mutations and/or to grow to tumours after the induction of appropriate mutations.

Otteneder and Lutz (1999) have reviewed the quantitative relationship between DNA adducts and tumour incidence in liver for 27 chemicals in 2-year bioassays. In rat liver, the calculated adduct concentration ‘responsible’ for a 50% hepatocellular tumour incidence (among the animals that would not have developed tumours without the treatment) spanned from 53 to 2083 adducts per 10^8^ nucleotides (determined by various methods, mostly ^32^P-postlabelling). In our study, 39–56 adducts per 10^8^ nucleotides were detected in the liver of the various experimental groups receiving a *Brassica*-containing diet. Thus, the adduct level might be sufficient to detectably enhance the tumour incidence, which of course has to be verified experimentally.

Although our animals had free access to standard laboratory feed, they consumed large amounts of *Brassica* vegetable, corresponding to nearly 10% of their body weight per day. This resulted in a high intake of nGBS, in the range of 2 mg per animal per day (in the broccoli-fed groups), or 6 mg per kg body weight per day. Human intake is much lower, although some populations may consume GL-producing plants (such as cabbage, broccoli, cauliflower, kohlrabi, pak choi, radish, rocket salad, cress and mustard) nearly every day. The average daily intake of nGBS has been estimated to 0.5 and 0.3 mg/day per person for some Danish and Finnish populations, respectively (Vang and Dragsted 1996). However, the variation may be very large depending on food preferences and modes of preparation. Some foods may contain very high levels of nGBS. For example, we have found up to 50 mg nGBS per 100 g fresh matter in pak choi (Wiesner et al. 2013). GLs are often associated with chemoprevention and anti-carcinogenicity, rather than with genotoxicity and possible carcinogenicity, aspects investigated in the present study. In particular, breakdown products of various GLs, for example sulforaphane formed from glucoraphanin, can activate the NRF2 transcription factor (IARC 2004; Lippmann et al. 2014; Zhang et al. 1994). This activation leads to the enhanced expression of various enzymes of detoxification. As a result, the carcinogenic effect of aflatoxin B_1_ and other chemicals can be strongly diminished. It is probable that enzyme induction also occurred in our study in the animals fed *Brassica* vegetables. Evidently, this induction was not sufficient to fully protect against DNA adduct formation by breakdown products of nGBS.

It is important to notice that not all GLs are equal. We have shown that nGBS is a potent genotoxicant in bacteria and mammalian cells in culture (Baasanjav-Gerber et al. 2011a). In the present study we demonstrated that natural levels present in *Brassica* vegetables are able to induce substantial levels of DNA adducts in animals. Adducts formed by other GLs have not been detected in the animal model used. Consequentially, it will be important to investigate possible impacts of the adduct formation by nGBS, such as carcinogenesis.

### Epilogue

After completion of this study, we chemically prepared *N*^2^-(1-MIM)-dG, *N*^6^-(1-MIM)-dA (Schumacher et al. 2012). We verified the structure of these standards by nuclear magnetic resonance (NMR) analysis. We devised and validated a UPLC–MS/MS method, using isotopically labelled internal standards, for their unambiguous detection and quantification in biological materials (Schumacher et al. 2012). In addition, we raised a polyclonal antiserum against 1-MIM DNA adducts for the immunohistochemical localisation of the adducts in tissue sections (Ehlers et al. 2015; Schumacher et al. 2014).

Early in this project, we had detected that incubation of nGBS with myrosinase leads to the formation of intermediates able to form DNA adducts and mutations in bacterial test systems (Baasanjav-Gerber et al. 2011b). Others had identified 1-MIM alcohol and 1-MIM nitrile as the first relatively stable degradation products formed from nGBS (1-MIM GL) by the action of myrosinase in aqueous solution, with evidence for the formation of short-lived 1-MIM isothiocyanate as an intermediate (Hanley et al. 1990). We chemically prepared 1-MIM alcohol and 1-MIM nitrile. They demonstrated negligible direct DNA reactivity or mutagenicity, suggesting that the isothiocyanate was the actual genotoxicant formed in the nGBS/myrosinase model. However, we detected that 1-MIM alcohol can be activated to a potent mutagen by mammalian sulphotransferases (SULTs) (Glatt et al. 2011). The DNA adduct patterns formed by 1-MIM alcohol/SULT in various models (cell-free, cells in culture and animals) were indistinguishable from those formed by nGBS/myrosinase (Schumacher et al. 2012, 2014). We suspect that the reactive species, 1-MIM isothiocyanate and 1-MIM sulphate, respectively, form the adducts via the identical benzylic (resonance-stabilised) cation.

Up to date, our new methods for the detection of 1-MIM DNA adducts, isotope-dilution UPLC–MS/MS and immunohistochemistry, were primarily employed in animal studies using individual compounds (nGBS or 1-MIM alcohol) as test materials (Budnowski et al. 2015; Ehlers et al. 2015; Gronke et al. 2019; Schumacher et al. 2014). Mice, rather than rats, were used due to their smaller body weight to reduce the quantities of test materials required. In an additional study, pak choi powder (prepared from lyophilised sprouts) and total GLs extract were mixed to the feed in a mouse study (up to 8-day treatment and up to 16-day follow-up under standard diet) (Wiesner-Reinhold et al. 2019). The main messages: (1) 1-MIM DNA adducts were detected in all models; (2) the tissue distribution of the adduct varied substantially depending, on the test material, the time of analysis and the presence or absence of intestinal bacteria; (3) adducts in gut were eliminated rapidly via cell renewal; (4) in other tissues (with low cell turnover), adducts were rather persistent and they accumulated after repeated dosing. Upregulation of DNA damage response, e.g. p53 accumulation and/or increased expression of p53-dependent genes was a common effect of treatment with GBS or 1-MIM alcohol (Ehlers et al. 2015; Schumacher et al. 2014; Wiesner-Reinhold et al. 2019). However, we found no indications for xenometabolic adaptation (decreased activation/increased detoxification of the test material) after repeated dosing. This conclusion was primarily inferred from the time courses of adducts in haemoglobin and serum albumin (Barknowitz et al. 2014; Wiesner-Reinhold et al. 2019), as protein adducts, in contrast to many DNA adducts, are not actively repaired in general. However, all these studies were conducted with individual test compounds (1-MIM GL or 1-MIM alcohol) or a matrix containing particularly high levels of 1-MIM-GL (pak choi sprouts elicited with methyl jasmonate, mimicking an attack by chewing and phloem-sucking insects). Unfortunately, we had no opportunity to monitor the time courses of the levels of DNA adduct in the present study, in which broccoli and cauliflower were fed to rats. However, the presence of relatively high levels of adducts in many tissues at the end of the 5-week feeding period indicates that any constitutive or inducible defence was insufficient to prevent DNA damage.

It would be useful for risk assessment to monitor 1-MIM adducts in humans. Such studies are under way in our laboratories. For the moment, we recommend using cultivars of *Brassica* vegetables, growth conditions and/or food preparations that keep the level of 1-MIM-GL low.

## Supplementary Information

Below is the link to the electronic supplementary material.

Tables on body weight development of rats, daily food intake (standard laboratory chow and Brassica vegetable), patterns and levels of GLs in vegetable batches used, calculated intake of total GLs and nGBS (DOCX 44 kb)

## Abbreviations

ACPI: Atmospheric pressure chemical ionisation
CID: Collision-induced dissociation
cv.: Cultivar
GBS: Glucobrassicin (3-indolylmethyl glucosinolate)
GL: Glucosinolate
HPLC: High-performance liquid chromatography
1-MIM: 1-Methoxy-3-indolylmethyl
MRM: Multiple reaction monitoring
MS: Mass spectrometry
*m/z*: Mass-to-charge ratio
nGBS: Neoglucobrassicin (1-methoxy-3-indolylmethyl glucosinolate)
NMR: Nuclear magnetic resonance
RAL: Relative adduct level
UPLC: Ultra-performance liquid chromatography
