# Supplementary material for: Feeding Brassica vegetables to rats leads to the formation of characteristic DNA adducts (from 1-methoxy-3-indolylmethyl glucosinolate) in many tissues
Source: Arch Toxicol. 2022 Jan 7;96(3):933–44. doi: 10.1007/s00204-021-03216-8 (PMC8850215; doi:10.1007/s00204-021-03216-8)
Supplement: Supplementary file 1 — Tables on body weight development of rats, daily food intake (standard laboratory chow and Brassica vegetable), patterns and levels of GLs in vegetable batches used, calculated intake of total GLs and nGBS (DOCX 44 kb) [file 204_2021_3216_MOESM1_ESM.docx]

# Feeding rats with *Brassica* vegetables leads to the formation of characteristic DNA adducts (from 1-methoxy-3-indolylmethyl glucosinolate) in many tissues

##### Supporting Information

**Hansruedi Glatt ^1,2^, Wolfram Engst ^1^, Simone Florian ^1^, Monika Schreiner ^3^, Chimgee Baasanjav-Gerber ^1^**

^1^ German Institute of Human Nutrition, Potsdam-Rehbrücke, 14558 Nuthetal, Germany

^2^ Department Food Safety, Federal Institute of Risk Assessment, Max-Dohrn-Strasse 8-10, 10589 Berlin, Germany

^3^ Leibniz Institute of Vegetable and Ornamental Crops, 14979 Grossbeeren, Germany

## * Corresponding author:

Prof. Hansruedi Glatt

Fichtestrasse 12

10967 Berlin, Germany

E-mail: glatt@dife.de

Phone: +49-30-691 6846

##

**Abbreviations**
GL, glucosinolate; GBS, glucobrassicin; GER, glucoerucin; GIB, glucoiberin; GRA, glucoraphanin; 4-MeO-GBS, 4-methoxyglucobrassicin; nGBS, neoglucobrassicin; 4-OH-GBS, 4-hydroxyglucobrassicin; SIN, sinigrin.

**Table S1.** Body weight development of the rats in experiment I.

| Age (weeks) | Body weight (g) | | | | | | | |
| --- | --- | --- | --- | --- | --- | --- | --- | --- |
|  | Rat 1 (control) | Rat 2 | Rat 3 | Rat 4 | Rat 5 | Rat 6 | Mean ± SD of rats 2–6 | |
| 6 | 217 | 231 | 216 | 233 | 204 | 204 | 218 ± | 14 |
| 7 | 278 | 288 | 279 | 285 | 263 | 263 | 276 ± | 12 |
| 8 | 335 | 346 | 344 | 346 | 323 | 318 | 335 ± | 14 |
| 9 | 388 | 387 | 396 | 396 | 375 | 364 | 384 ± | 14 |
| 10 | 435 | 436 | 451 | 442 | 413 | 405 | 429 ± | 20 |
| 11 | 477 | 460 | 487 | 480 | 454 | 439 | 464 ± | 19 |

**Table S2.** Consumption of standard chow in experiment I.

| Period (age of rats, weeks) | Standard chow (g/w) | | | | | | |
| --- | --- | --- | --- | --- | --- | --- | --- |
|  | Rat 1 (control) | Rat 2 | Rat 3 | Rat 4 | Rat 5 | Rat 6 | Mean ± SD of rats 2–6 |
| 6–7 | 182 | 162 | 268 | 175 | 172 | 157 | 187 ± 46 |
| 7–8 | – ^a^ | – ^a^ | – ^a^ | – ^a^ | – ^a^ | – ^a^ | – ^a^ |
| 8–9 | 214 | 176 | 202 | 169 | 189 | 180 | 183 ± 13 |
| 9–10 | 217 | 180 | 208 | 149 | 189 | 189 | 183 ± 22 |
| 10–11 | 204 | 173 | 215 | 185 | 192 | 187 | 190 ± 16 |

^a^ Consumption of standard chow was not determined in the second week of the experiment (age week 7–8).

**Table S3.** Consumption of broccoli in experiment I.

| Period (age of rats, weeks) | Broccoli (g/d, mean in indicated period) | | | | | |
| --- | --- | --- | --- | --- | --- | --- |
|  | Rat 2 | Rat 3 | Rat 4 | Rat 5 | Rat 6 | Mean ± SD of rats 2–6 |
| 6–7 | 24 | 20 | 33 | 25 | 28 | 26 ± 5 |
| 7–8 | 29 | 18 | 42 | 30 | 19 | 28 ± 9 |
| 8–9 | 34 | 23 | 61 | 33 | 23 | 35 ± 16 |
| 9–10 | 37 | 25 | 77 | 40 | 22 | 40 ± 22 |
| 10–11 | 43 | 30 | 97 | 55 | 34 | 52 ± 27 |
| Total (6–11) | 34 | 24 | 65 | 38 | 26 | 36 ± 11 |

**Table S4.** GL levels in broccoli fed in experiment I.

| Period (age of rats, weeks) ^a^ | Dry matter (% of fresh matter) | GL level (mg per 100 g fresh matter) | | | | | | | | |
| --- | --- | --- | --- | --- | --- | --- | --- | --- | --- | --- |
|  |  | GIB | GER | GRA | 4-OH-GBS | GBS | 4-MeO-GBS | nGBS | Total |  |
| 9–10 | 10.2 | 2.04 | n.d. | 12.67 | 0.42 | 8.97 | 1.14 | 7.16 | 32.39 |  |
| 10–11 | 10.8 | 1.81 | n.d. | 11.99 | 0.52 | 8.06 | 1.12 | 3.14 | 26.64 |  |

^a^ GL levels were not determined in the batches of broccoli fed in the first three weeks of the experiment.

n.d. not detectable (limit of detection 0.04 mg per 100 g fresh matter).

**Table S5.** Intake of GLs in broccoli-fed rats of experiment I.

| Period (age of rats, weeks) | GLs intake (mg/d/animal, mean ± SD^a^) | | | | | |
| --- | --- | --- | --- | --- | --- | --- |
|  | Rat 2 | Rat 3 ^a^ | Rat 4 ^a^ | Rat 5 ^a^ | Rat 6 ^a^ | Rats 2–6 ^b^ |
| Total GLs | | | | | | |
| 9–10 | 11.8 ± 2.2 | 8.2 ± 1.9 | 24.8 ± 6.7 | 13.0± 2.0 | 7.2 ± 2.1 | 13.0 ± 7.0 |
| 10–11 | 12.1 ± 3.1 | 8.3 ± 2.5 | 26.6 ± 7.8 | 15.2 ± 5.2 | 9.5 ± 4.5 | 14.3 ± 7.3 |
| 9–11 ^b^ | 12.0 ± 2.7 | 8.3 ± 2.2 | 25.8 ± 7.1 | 14.2 ± 4.1 | 8.5 ± 3.8 | 13.7 ± 7.2 |
| *nGBS* | | | | | | |
| 9–10 | 2.6 ± 0.5 | 1.8 ± 0.4 | 5.5 ± 1.5 | 2.9 ± 0.4 | 1.6 ± 0.5 | 2.9 ± 1.6 |
| 10–11 | 1.7 ± 0.8 | 1.1 ± 0.4 | 3.6 ± 0.9 | 2.1 ± 1.0 | 1.3 ± 0.6 | 2.0 ± 1.0 |
| 9–11 ^b^ | 2.1 ± 0.8 | 1.4 ± 0.5 | 4.4 ± 1.5 | 2.4 ± 0.9 | 1.4 ± 0.5 | 2.4 ± 1.2 |

^a^ For the individual animals, SD refers to the daily variation. For the mean of all broccoli-fed animals (last column), it refers to the variation among the animals (using the mean intake in the indicated period as the statistical unit).

^b^ GLs were not determined in the initial three weeks of the experiment. Supposing that the daily intake of GLs was similar during the entire experiment (35 d), the total GL and nGBS intake was approximately 35-fold higher than the values given in the last line of each table block. Thus, the total intake of GLs in the entire experimental period is estimated to 419, 289, 903, 498 and 296 mg for the individual animals (481 ± 252, mean ± SD). The corresponding values for nGBS are 74, 50, 154, 85 and 50 (83 ± 43, mean ± SD).

**Table S6.** Body weight development of the rats in experiment II.

| Age (weeks) | Body weight (g, mean ± SD of the 6 animals used in each group) ^a^ | | | |
| --- | --- | --- | --- | --- |
|  | Group 1 (rats 1–6, control) | Group 2 (rats 7–12, raw broccoli) | Group 3 (rats 13–18, steamed broccoli) | Group 4  (rats 19–24, raw cauliflower) |
| 7 | 253 ± 11 | – | – | – |
| 8 | 319 ± 34 | 335 ± 22 | 346 ± 16 | 347 ± 26 |
| 9 | 339 ± 15 | 399 ± 42 | 396 ± 22 | 371 ± 28 |
| 10 | 373 ± 17 | 414 ± 23 | 426 ± 24 | 403 ± 30 |
| 11 | 420 ± 23 | 443 ± 24 | 454 ± 26 | 433 ± 33 |
| 12 | – | 480 ± 26 | 493 ± 33 | 473 ± 40 |

^a^ Animals of groups 2–4 were purchased at the same time and randomly grouped. Animals of the control group were purchased 3 weeks later.

**Table S7.** Consumption of standard chow in experiment II.

| Period (age of rats, weeks) | Standard chow (g/w, mean ± SD of the 6 animals used in each group) | | | |
| --- | --- | --- | --- | --- |
|  | Group 1 (rats 1–6, control) | Group 2 (rats 7–12, raw broccoli) | Group 3 (rats 13–18, steamed broccoli) | Group 4  (rats 19–24, raw cauliflower) |
| 8–9 | 177 ± 5 | 178 ± 15 | 192 ± 19 | 164 ± 12 |
| 9–10 | 180 ± 9 | 180 ± 12 | 196 ± 15 | 188 ± 17 |
| 10–11 | 180 ± 9 | 175 ± 14 | 186 ± 14 | 185 ± 19 |
| 11–12 | 211 ± 7 | 206 ± 16 | 219 ± 14 | 216 ± 17 |
| 12–13 | – ^a^ | – ^a^ | – ^a^ | – ^a^ |

^a^ Consumption of standard chow was not determined in the last week of the experiment (age week 12–13).

**Table S8.** Consumption of *Brassica* vegetable in experiment II.

| Period (age of rats, weeks) | *Brassica* vegetables (g/d, mean ± SD ^a^) | | |
| --- | --- | --- | --- |
|  | Group 2 (rats 7–12, raw broccoli) | Group 3 (rats 13–18, steamed broccoli) | Group 4  (rats 19–24, raw cauliflower) |
| 8–9 | 41 ± 9 | 46 ± 12 | 38 ± 18 |
| 9–10 | 46 ± 10 | 46 ± 13 | 42 ± 25 |
| 10–11 | 48 ± 12 | 47 ± 14 | 37 ± 19 |
| 11–12 | 49 ± 12 | 43 ± 10 | 36 ± 20 |
| 12–13 | 59 ± 10 | 56 ± 12 | 44 ± 25 |
| Total (8–13) | 48 ± 7 | 47 ± 5 | 39 ± 4 |

^a^ SD refers to the variation among the animals (n = 6), using the mean daily intake in the indicated period as the statistical unit.

**Table S9.** GL levels in *Brassica* vegetables fed in experiment II.

| Period (age of rats, weeks) | Dry matter (% of fresh matter) | GL level (mg per 100 g fresh matter) | | | | | | | | |  |
| --- | --- | --- | --- | --- | --- | --- | --- | --- | --- | --- | --- |
|  |  | GIB | GER | SIN | GRA | 4-OH-GBS | GBS | 4-MeO-GBS | nGBS | Total |  |
| *Broccoli* ^a^ | | | | | | | | | | | |
| 8–8.5 | 11.8 | 2.83 | 0.00 | n.d | 17.68 | 0.44 | 8.39 | 0.96 | 7.45 | 37.75 |  |
| 8.5–9 | 10.6 | 2.56 | 0.82 | n.d | 8.82 | 0.30 | 3.42 | 1.15 | 5.04 | 22.11 |  |
| 9–9.5 ^b^ | 10.4 | 1.51 | 0.54 | n.d | 7.04 | 0.49 | 2.83 | 0.94 | 1.43 | 14.79 |  |
| 9.5–10 ^b^ | 10.0 | 0.45 | 0.00 | n.d | 2.54 | 0.20 | 5.37 | 0.64 | 0.54 | 9.74 |  |
| 10–11 | 11.6 | 2.57 | 0.00 | n.d | 17.83 | 1.39 | 10.73 | 1.49 | 7.30 | 41.32 |  |
| 11–12 ^b^ | 10.9 | 0.91 | 0.00 | n.d | 5.70 | 0.29 | 1.37 | 0.46 | 0.64 | 9.36 |  |
| 12–12.5 | 11.7 | 2.99 | 0.00 | n.d | 15.61 | 0.35 | 5.71 | 0.80 | 5.34 | 30.81 |  |
| 12.5–13 ^b^ | 12.0 | 2.73 | 0.00 | n.d | 9.39 | 0.35 | 5.74 | 0.58 | 1.53 | 20.32 |  |
| *Cauliflower* | | | | | | | | | | | |
| 8–9 | 7.0 | 3.30 | n.d | 1.30 | 0.16 | 0.42 | 3.16 | 0.21 | 0.34 | 8.88 |  |
| 9–10 | 7.3 | 1.08 | n.d | 0.92 | 0.05 | 0.62 | 2.20 | 0.31 | 0.33 | 5.52 |  |
| 10–11 | 7.9 | 2.57 | n.d | 3.01 | 0.38 | 0.44 | 3.67 | 0.24 | 0.50 | 10.81 |  |
| 11–12 | 7.0 | 1.60 | n.d | 0.58 | 0.16 | 0.47 | 1.87 | 0.32 | 0.22 | 5.22 |  |
| 12–12.5 | 7.6 | 2.13 | n.d | 2.34 | 0.11 | 0.55 | 3.51 | 0.36 | 1.42 | 10.42 |  |
| 12.5–13 | 7.2 | 3.22 | n.d | 3.15 | 0.13 | 0.47 | 7.29 | 0.62 | 3.79 | 18.67 |  |

^a^ GL levels were only determined in raw broccoli. However, control experiments indicated that the level was practically unaffected by the steaming regimen used (100°C for 15 min).

^b^ Broccoli from food shops. It had to be used, as the animals consumed more broccoli than we had produced ourselves.

n.d. not detectable (limit of detection 0.04 mg per 100 g fresh matter).

**Table S10.** Intake of total GLs in rats receiving *Brassica*-containing diets in experiment II.

| Period (age of rats, weeks) | Total GLs (mg/d/animal, mean ± SD ^a^) | | | | | | |
| --- | --- | --- | --- | --- | --- | --- | --- |
|  | *Raw broccoli* | | | | | | |
|  | Rat 7 | Rat 8 | Rat 9 | Rat 10 | Rat 11 | Rat 12 | Rats 7–12 |
| 8–9 | 13.9 ± 2.9 | 12.0 ± 4.1 | 13.0 ± 5.0 | 8.7 ± 3.6 | 16.4 ± 6.4 | 16.6 ± 3.8 | 13.4 ± 3.0 |
| 9–10 | 7.7 ± 0.9 | 9.4 ± 1.7 | 7.8 ± 0.8 | 5.4 ± 0.7 | 10.7 ± 2.1 | 10.3 ± 2.6 | 8.5 ± 2.0 |
| 10–11 | 11.7 ± 7.6 | 12.1 ± 7.9 | 10.6 ± 6.7 | 6.4 ± 4.0 | 13.5 ± 8.3 | 15.2 ± 10.7 | 11.6 ± 3.0 |
| 11–12 | 14.1 ± 7.7 | 14.1 ± 6.4 | 17.8 ± 9.0 | 8.0 ± 4.3 | 17.4 ± 8.7 | 18.2 ± 9.8 | 14.9 ± 3.9 |
| 12–13 | 15.0 ± 5.1 | 15.6 ± 4.8 | 17.6 ± 6.0 | 10.4 ± 5.5 | 16.3 ± 6.5 | 16.9 ± 7.3 | 15.3 ± 2.6 |
| Total (8-13) | 12.5 ± 3.0 | 12.7 ± 2.4 | 13.3 ± 4.4 | 7.8 ± 2.0 | 14.8 ± 2.8 | 15.5 ± 3.1 | 12.8 ± 2.7 |
|  | *Steamed broccoli* | | | | | | |
|  | Rat 13 | Rat 14 | Rat 15 | Rat 16 | Rat 17 | Rat 18 | Rats 13–18 |
| 8–9 | 11.4 ± 3.7 | 10.4 ± 4.0 | 14.2 ± 5.5 | 15.8 ± 5.3 | 18.4 ± 4.5 | 19.7 ± 4.6 | 15.0 ± 3.7 |
| 9–10 | 7.5 ± 3.9 | 5.4 ± 4.1 | 7.7 ± 5.8 | 8.2 ± 5.6 | 11.1 ± 5.4 | 11.9 ± 5.0 | 8.6 ± 2.4 |
| 10–11 | 9.6 ± 3.1 | 7.4 ± 2.8 | 8.7 ± 5.2 | 12.0 ± 5.4 | 15.7 ± 4.9 | 16.3 ± 4.0 | 11.6 ± 3.7 |
| 11–12 | 15.0 ± 2.4 | 10.1 ± 1.7 | 14.5 ± 3.7 | 17.0 ± 3.6 | 20.1 ± 3.9 | 19.2 ± 3.9 | 16.0 ± 3.7 |
| 12–13 | 13.0 ± 1.3 | 10.5 ± 1.0 | 12.0 ± 1.6 | 14.2 ± 2.4 | 17.7 ± 1.0 | 17.5 ± 1.9 | 14.2 ± 2.9 |
| Total (8-13) | 11.3 ± 2.9 | 8.8 ± 2.3 | 11.4 ± 3.1 | 13.4 ± 3.5 | 16.6 ± 3.5 | 16.9 ± 3.1 | 13.1 ± 3.2 |
|  | *Raw cauliflower* | | | | | | |
|  | Rat 19 | Rat 20 | Rat 21 | Rat 22 | Rat 23 | Rat 24 | Rats 19–24 |
| 8–9 | 1.9 ± 0.4 | 5.8 ± 1.4 | 2.9 ± 0.5 | 2.4 ± 0.8 | 2.0 ± 0.5 | 5.0 ± 0.6 | 3.3 ± 1.6 |
| 9–10 | 1.2 ± 0.5 | 4.3 ± 0.8 | 1.8 ± 1.1 | 1.6 ± 0.4 | 1.2 ± 0.3 | 3.7 ± 0.4 | 2.3 ± 1.4 |
| 10–11 | 2.3 ± 0.7 | 7.0 ± 1.2 | 3.5 ± 1.0 | 2.8 ± 0.6 | 2.3 ± 0.5 | 6.3 ± 0.7 | 4.0 ± 2.1 |
| 11–12 | 1.9 ± 1.1 | 6.5 ± 2.8 | 2.8 ± 1.5 | 2.1 ± 1.0 | 2.0 ± 0.9 | 4.6 ± 1.6 | 3.3 ± 1.9 |
| 12–13 | 3.3 ± 2.1 | 12.6 ± 6.1 | 6.5 ± 4.3 | 3.6 ± 2.7 | 5.0 ± 6.1 | 9.8 ± 5.7 | 6.8 ± 3.7 |
| Total (8-13) | 2.1 ± 0.8 | 7.2 ± 3.2 | 3.5 ± 1.8 | 2.5 ± 0.8 | 2.5 ± 1.5 | 5.9 ± 2.4 | 4.0 ± 2.1 |

^a^ For the individual animals, SD refers to the daily variation. For the mean of all animals of a group (last column), it refers to the variation among the animals (using the mean intake in the indicated period as the statistical unit).

**Table S11.** Intake of nGBS in rats receiving *Brassica*-containing diets in experiment II.

| Period (age of rats, weeks) | nGBS (mg/d/animal, mean ± SD ^a^) | | | | | | |
| --- | --- | --- | --- | --- | --- | --- | --- |
|  | *Raw broccoli* | | | | | | |
|  | Rat 7 | Rat 8 | Rat 9 | Rat 10 | Rat 11 | Rat 12 | Rats 7–12 |
| 8–9 | 2.8 ± 0.4 | 2.4 ± 0.7 | 2.6 ± 0.9 | 1.8 ± 0.7 | 3.3 ± 1.1 | 3.4 ± 0.6 | 2.7 ± 0.6 |
| 9–10 | 1.4 ± 0.6 | 1.7 ± 0.9 | 1.4 ± 0.6 | 0.9 ± 0.4 | 1.9 ± 1.0 | 1.9 ± 1.1 | 1.5 ± 0.4 |
| 10–11 | 1.6 ± 1.7 | 1.7 ± 1.8 | 1.5 ± 1.5 | 0.9 ± 0.9 | 1.9 ± 1.9 | 2.2 ± 2.4 | 1.6 ± 0.4 |
| 11–12 | 2.4 ± 1.5 | 2.3 ± 1.4 | 3.0 ± 1.8 | 1.3 ± 0.9 | 2.9 ± 1.8 | 3.0 ± 2.0 | 2.5 ± 0.6 |
| 12–13 | 2.0 ± 0.7 | 2.3 ± 0.9 | 2.4 ± 1.0 | 1.4 ± 0.7 | 2.3 ± 1.1 | 2.2 ± 0.8 | 2.1 ± 0.4 |
| Total (8-13) | 2.0 ± 0.6 | 2.1 ± 0.4 | 2.2 ± 0.7 | 1.3 ± 0.4 | 2.5 ± 0.6 | 2.6 ± 0.6 | 2.1 ± 0.5 |
|  | *Steamed broccoli* | | | | | | |
|  | Rat 13 | Rat 14 | Rat 15 | Rat 16 | Rat 17 | Rat 18 | Rats 13–18 |
| 8–9 | 2.3 ± 0.6 | 2.1 ± 0.7 | 2.9 ± 1.0 | 3.2 ± 1.0 | 3.8 ± 0.7 | 4.0 ± 0.7 | 3.1 ± 0.8 |
| 9–10 | 1.3 ± 0.6 | 0.9 ± 0.4 | 1.4 ± 0.8 | 1.5 ± 0.8 | 2.0 ± 1.0 | 2.2 ± 1.2 | 1.5 ± 0.5 |
| 10–11 | 1.3 ± 1.4 | 1.0 ± 1.1 | 1.2 ± 1.3 | 1.7 ± 1.9 | 2.3 ± 2.5 | 2.3 ± 2.5 | 1.7 ± 0.6 |
| 11–12 | 2.0 ± 1.2 | 1.3 ± 0.8 | 2.0 ± 1.2 | 2.3 ± 1.5 | 2.5 ± 2.0 | 2.5 ± 1.5 | 2.1 ± 0.5 |
| 12–13 | 1.7 ± 0.8 | 1.4 ± 0.7 | 1.6 ± 0.8 | 1.9 ± 1.0 | 2.2 ± 2.0 | 2.3 ± 1.2 | 1.9 ± 0.4 |
| Total (8-13) | 1.7 ± 0.4 | 1.4 ± 0.5 | 1.8 ± 0.7 | 2.1 ± 0.7 | 2.6 ± 0.7 | 2.7 ± 0.6 | 2.0 ± 0.6 |
|  | *Raw cauliflower* | | | | | | |
|  | Rat 19 | Rat 20 | Rat 21 | Rat 22 | Rat 23 | Rat 24 | Rats 19–24 |
| 8–9 | 0.07 ± 0.01 | 0.22 ± 0.05 | 0.11 ± 0.02 | 0.09 ± 0.03 | 0.08 ± 0.02 | 0.19 ± 0.02 | 0.13 ± 0.06 |
| 9–10 | 0.07 ± 0.03 | 0.26 ± 0.05 | 0.11 ± 0.07 | 0.09 ± 0.02 | 0.07 ± 0.02 | 0.22 ± 0.02 | 0.14 ± 0.08 |
| 10–11 | 0.10 ± 0.03 | 0.32 ± 0.06 | 0.16 ± 0.05 | 0.13 ± 0.03 | 0.11 ± 0.02 | 0.29 ± 0.03 | 0.19 ± 0.10 |
| 11–12 | 0.24 ± 0.18 | 0.82 ± 0.06 | 0.35 ± 0.24 | 0.27 ± 0.17 | 0.24 ± 0.16 | 0.55 ± 0.33 | 0.41 ± 0.23 |
| 12–13 | 0.61 ± 0.47 | 2.31 ± 1.52 | 1.18 ± 0.98 | 0.67 ± 0.60 | 0.97 ± 1.28 | 1.80 ± 1.34 | 1.26 ± 0.67 |
| Total (8-13) | 0.22 ± 0.23 | 0.79 ± 0.88 | 0.38 ± 0.46 | 0.25 ± 0.25 | 0.29 ± 0.39 | 0.61 ± 0.68 | 0.42 ± 0.23 |

^a^ For the individual animals, SD refers to the daily variation. For the mean of all animals of a group (last column), it refers to the variation among the animals (using the mean intake in the indicated period as the statistical unit).
